# Modelling Energy Demands of Cross-Country Tests in 2-Star to 5-Star Eventing Competitions

**DOI:** 10.3390/ani15121775

**Published:** 2025-06-17

**Authors:** Anna M. Liedtke, Hans Meijer, Stephanie Horstmann, Caroline von Reitzenstein, Insa Rump, Katharina Kirsch

**Affiliations:** 1 German Olympic Equestrian Committee (DOKR), Freiherr-von-Langen-Str. 15, 48231 Warendorf, Germany; aliedtke@fn-dokr.de (A.M.L.); hmeijer@fn-dokr.de (H.M.); creitzenstein@fn-dokr.de (C.v.R.);; 2 Institute of Animal Welfare, Animal Behaviour and Laboratory Animal Science, Freie Universität Berlin, Königsweg 67, 14163 Berlin, Germany

**Keywords:** eventing, energy expenditure, cross-country, internal load, external load, metabolic power, anaerobic demand, heart rate, cost of transport, GPS data

## Abstract

Eventing is an Olympic equestrian discipline comprising dressage, cross-country, and show jumping, with the cross-country phase imposing the greatest physical demands on horses. This study presents a composite model to estimate energy expenditure during the cross-country phase, integrating physiological data (heart rate-derived $VO_{2}$ and lactate-based anaerobic estimates) with external workload indicators (GPS-derived speed, elevation, and course complexity). Model development was based on 691 rides from 256 horses across 232 events at 2-star to 5-star competition levels. The analysis showed that terrain, speed variability, and acceleration, largely shaped by course design, significantly affect energy expenditure. Aerobic and anaerobic contributions to power output varied by speed, format, and competition level. The model explained 29% of variance in power output and 91% when accounting for random effects, demonstrating the influence of both external and individual factors. Short-format events exhibited higher anaerobic contributions than long-format events. While the competition level had a modest effect, it reflected increasing technical difficulty and jump size. These findings underline the importance of incorporating both physiological responses and course characteristics in energy assessments. The model supports more targeted conditioning, enhances performance monitoring, and contributes to improved equine welfare by providing a more accurate understanding of workload in cross-country competitions.

## Simple Summary

Eventing is an Olympic equestrian sport that includes dressage, cross-country, and show jumping. Among these, the cross-country phase is the most physically demanding for the horse. This study developed a new way to estimate how much energy horses use during the cross-country phase by combining data on their heart rate and blood lactate levels with information about the course, like speed, terrain, and level of difficulty. The model was based on 691 rides from over 256 horses competing at various international levels. It showed that course design, especially hills, changes in speed, and turns, strongly affects how hard horses have to work. The study also found that shorter competitions push horses more into anaerobic (high-intensity) effort, while longer ones rely more on steady aerobic work. This model helps riders, trainers, and veterinarians better understand the demands placed on horses during cross-country, allowing for more tailored fitness training and improved horse welfare.

## 1. Introduction

Eventing is considered the most comprehensive of the Olympic equestrian disciplines. Representing the ultimate test of versatility and athleticism, it combines dressage, cross-country, and show jumping, the triathlon of equestrian sports. The cross-country phase imposes particularly high physical and physiological demands, requiring sustained galloping, rapid directional changes, and complex jumping efforts across varied terrain. Inadequate preparation can increase the risk of injury and compromise equine welfare [1]. Recent studies have emphasised the significance of incorporating physiological and biomechanical indicators to detect fatigue and optimise conditioning in event horses [1,2]. However, the precise demands of a given cross-country course are not yet fully understood. Only a small number of studies have attempted to quantify the overall energetic cost of cross-country competitions [3]. In response to this need, the present study aimed to develop a composite model that estimates the energy demands of cross-country tests by integrating internal physiological responses (heart rate-derived $VO_{2}$ and lactate-based anaerobic estimates) with external workload indicators (GPS-derived speed, changes in elevation and acceleration). The model was developed using a database of 691 cross-country rides containing heart rate (HR), GPS, and lactate data, and applied to a broader dataset of 1978 rides with GPS data to compare energy demands across competition levels (2-star to 5-star) and formats (CCI-L and CCI-S). This approach seeks to provide a more holistic measure of equine workload and to support individualised training strategies and risk management.

The use of tracking technology in equestrian sports has seen a marked increase in recent years, with the primary objective of quantifying the physical demands associated with training and competition. In order to develop targeted training programmes, it is imperative to ascertain the quantity of energy expended during competitive events and to determine the distribution of this energy between the aerobic and anaerobic energy systems. Several studies have described the physiological and biochemical responses of horses competing in the cross-country test of international eventing competitions. These responses have been based on GPS data, heart rate, or blood values [4,5,6,7,8].

Heart rate is one of the most accessible and widely used indicators of aerobic energy expenditure. This is due to the fact that it reflects the cardiovascular system’s effort to meet oxygen demands during physical exertion. Changes in speed, incline, acceleration, deceleration, directional changes, and jumping all influence oxygen consumption and are mirrored by HR fluctuations. However, HR is not only affected by biomechanical effort but also by environmental factors such as heat and humidity, as well as the individual horse’s health and fitness status. A close relationship has been demonstrated between HR and oxygen consumption ($VO_{2}$) in horses during exercise [9]. This relationship has been further quantified by Coenen et al. [10], who synthesised data from approximately 80 studies comprising 569 paired data points to model the estimation of $VO_{2}$ from HR, enabling practical assessment of aerobic load in equine performance contexts. In the present model, the maximum rate of oxygen consumption ($VO_{2}max$) is utilised to evaluate the contribution of the aerobic metabolism and the post-exercise blood lactate concentration to estimate the contribution of the anaerobic metabolism to meet energy demands.

Although aerobic metabolism accounts for the majority of the energy requirements during moderate to intense exercise, anaerobic metabolism contributes to energy production across all exercise intensities, with its relative contribution increasing as intensity rises. It is estimated that at exercise intensities ranging from 110 to 115% of $VO_{2}max$, the contribution of anaerobic metabolism to the total energy requirement ranges between 21.3 and 30% [11]. Blood lactate measurements serve as a valuable proxy for estimating this contribution, as lactate accumulates in muscle and blood when glycolytic production exceeds the capacity for lactate removal via oxidation [12]. However, lactate is not merely a byproduct, it functions as a substrate that can be oxidised by various tissues, including the heart and oxidative muscle fibres, thereby playing a role in energy supply and signalling adaptations [13]. Provided that lactate production does not exceed systemic clearance capacity, steady-state blood lactate concentration is maintained, typically between 2 and 8 mmol/L in humans [14]. However, in horses, the maximal intensity of exercise in which blood lactate production and clearance are in balance has been shown to be lower, not exceeding concentrations of 2 mmol/L, probably due to the higher proportion of skeletal muscle mass involved in exercise [15]. Beyond this threshold, an increase in the intensity of exercise results in an exponential rise in lactate levels due to an increased anaerobic contribution [16]. In the context of the cross-country phases of eventing, equines frequently attain blood lactate concentrations that surpass 20 mmol/L, underscoring the considerable anaerobic demands inherent in these events and the remarkable physiological plasticity of equine athletes [6].

While internal load provides important insights into the physiological response of the horse, it must be contextualised within the mechanical demands imposed by the course itself. One key determinant is the terrain. The degree of incline at which the exercise is performed exerts a substantial influence on energy expenditure. A substantial number of studies conducted on mammals, including equines, have shown that the total energetic cost of transport per unit distance remains constant and independent of velocity [17,18,19]. However, it has been observed to increase in response to increasing gradients and decrease in response to decreasing gradients [3]. As demonstrated in previous research, greater height differences have been shown to result in a significant increase in heart rate and post-exercise blood lactate concentrations [5]. The terrain of a cross-country course has been shown to have a significant impact on energy expenditure [20]. Schroter and Marlin [3] have developed a model for estimating the actual oxygen cost of transport in horses when running on variable gradient terrain. The model is based on empirical values obtained from horses exercising on a treadmill with an increasingly steep uphill gradient. It also incorporates predicted values derived from scaled data collected from humans running on various downhill gradients [3,21].

In addition to velocity and incline, there are other variables that have the capacity to exert an influence on the energetic demands of cross-country competitions. A preceding study [5] examined the physiological demands of cross-country competitions at varying levels. The study posited that competitions comprising a greater number of jumps per unit distance result in elevated heart rates and heightened blood lactate concentrations.

Despite the availability of physiological and external metrics, a unified approach to quantifying total workload in event horses has been lacking. By integrating internal and external load indicators, this study introduces a novel framework to characterise cross-country effort. The proposed model aims to enhance conditioning programmes and inform performance analysis and evidence-based training, ultimately improving welfare monitoring in the eventing horse population.

## 2. Materials and Methods

### 2.1. Horses and Competitions

A total of 394 skeletally mature horses of various levels of experience and performance were observed over the course of 1978 cross-country rides, which took place at 60 different venues across Europe at 464 different events between 2011 and 2025. To develop the energy cost model, only data from rides where GPS data, heart rate and post-exercise blood lactate concentrations were available were used. This subset consisted of data collected from 256 horses during 691 cross-country rides held at 32 different venues and across 232 events. These competitions were held under the rules of the Fédération Équestre Internationale (FEI) and ranged from preliminary (2-star) to the most demanding (5-star) level. Competitions included both short (CCI-S) and long (CCI-L) format competitions. The cross-country phase of the short format involves a shorter distance than the long format, yet the level of difficulty and the amount of efforts required is analogous to that of the long format within a given star level, thereby augmenting the intensity of the efforts. Also, for CCI-L competitions, the cross-country phase is scheduled before the show jumping phase, while for CCI-S competitions, the cross-country test may also, and is preferred to, take place on the last day after the show jumping and dressage phases. Only horses that completed the cross-country phase were included in the analysis. All horses belonged to riders who were qualified for the German national eventing squad, and the data were collected as part of the ‘Performance Monitoring Programme’ of the German Olympic Equestrian Committee (DOKR). The programme aims to promote the long-term health and performance of elite eventing horses by providing performance diagnostics and evidence-based advice on training.

### 2.2. Measurements During Cross-Country Competitions

Throughout the cross-country phase, heart rate and GPS data were recorded by either a heart rate sensor (Polar Equine T52H or H7, Polar Electro Oy, Kempele, Finland) and an additional GPS device (Equipilot, Fidelak GmbH, Kamen, Germany), or an integrated heart rate and GPS monitoring system (Equimetre, Arioneo, Paris, France). The heart rate and GPS data were recorded at a frequency of 1 Hz. Elevation data for the recorded GPS tracks were downloaded from ‘GPS Visualizer’ (https://www.gpsvisualizer.com/elevation accessed between 1 January 2020 and 31 March 2025). This open-source database provides Shuttle Radar Topography Mission (SRTM) and Light Detection and Ranging (LiDAR) data with an accuracy of one arcsecond (approximately 30 m). The vertical accuracy of SRTM data ranges between approximately 5 and 10 m, while the vertical accuracy of LiDAR data ranges between approximately 25 and 55 cm. For most venues, LiDAR data with a higher level of accuracy was available. Venous blood samples were collected 10 min after the conclusion of the cross-country phase. Blood samples were collected by venipuncture of the jugular vein using 3 mL plastic syringes and 21G needles. The lactate concentrations were determined immediately after the collection of the whole blood samples using a handheld photometer (Lactate Photometer Plus DP 110, Diaglobal, Berlin, Germany). These concentrations were subsequently confirmed in the in-house laboratory (Biosen C-Line, EKF Diagnostics, Barleben, Germany). Blood samples preserved for subsequent analysis within the facility’s in-house laboratory were stored in glucose/lactate haemolysing solution (EKF Diagnostics, Barleben, Germany), which ensures the stability of the sample over several days. Prior to 2021, data collection was conducted using the handheld photometer device only, as the Biosen was not acquired until late 2020. The lactate values obtained through the two methodologies were comparable and fell within the manufacturer-stated sensitivity and specificity ranges of the devices themselves.

### 2.3. Data Processing and Model Development

#### 2.3.1. Determination of Instantaneous Speed, Acceleration and Slope

For the cross-country phase, the instantaneous speed ($m\cdot s^{-1}$) was calculated as the distance (m) between subsequent GPS locations divided by the time difference (s) rather than using the speed given by the tracking devices. Due to discrepancies in the primary raw GPS data processing by the suppliers, it was necessary to apply uniform data transformation in order to ensure congruent utilisation of location data obtained from both devices. Varying degrees of filtering applied by the suppliers would have led to distortions of the acceleration and deceleration derived from pre-processed speed. Instantaneous acceleration ($m\cdot s^{-2}$) was calculated as the difference in speed between subsequent data points divided by the time difference. The slope of the course was calculated by dividing the difference in altitude between the subsequent data points by the distance.

#### 2.3.2. Estimating the Energy Cost of Cross-Country Courses over Ground of Varying Slope

The energetic cost of transport (COT) is usually measured as the rate at which oxygen is consumed per metre (expressed in $\mathrm{mL}\cdot {\mathrm{kg}}^{-1}\cdot m^{-1}$). Metabolic power is calculated as the product of the instantaneous energy cost (expressed in $J\cdot {\mathrm{kg}}^{-1}\cdot m^{-1}$) and speed ($m\cdot s^{-1}$). Thus, metabolic power is the amount of energy required per second per kilogram, which can be further reduced to $W\cdot {\mathrm{kg}}^{-1}$.

Running on the flat ground: The COT for running on flat ground ($COT_{path}$) was estimated according to [3]:

$$COT_{path}=0.123\;\mathrm{mL}\cdot {\mathrm{kg}}^{-1}\cdot m^{-1}$$

Running on ground of variable slope: The model proposed by Schroter and Marlin [3] was adapted for estimating the additional cost of running up and down variable slopes ($COT_{elev}$):

$$Uphill:\;COT_{elev}=1.561\cdot Slope$$

$$Downhill:\;COT_{elev}=1.561\cdot Slope+9.762\cdot Slope^{2}+14.0\cdot Slope^{3}$$

Accelerated and decelerated running: It was hypothesised that the frequent deceleration and acceleration that is required for technical-demanding cross-country courses may considerably increase the overall energetic requirements [5]. The model was thus extended to encompass the additional energetic cost of accelerated and decelerated running ($COT_{acc}$). During the process of acceleration, the centre of mass is shifted in the direction of motion. Conversely, during deceleration, the centre of mass is shifted in the opposite direction. According to [22], this suggests that the energy expenditure during accelerated running is equivalent to that during up-hill running at a constant speed up an equivalent slope. The overall acceleration ($g^{\prime }$) acting on the runner’s body was calculated as the vectorial sum of the forward acceleration ($a_{f}$) and the Earth’s acceleration of gravity (*g*), both assumed to be applied on the body’s centre of mass:

$$g^{\prime }=\sqrt[{}]{a_{f}^{2}+9.81^{2}}$$

The angle α between *g* and the ground was calculated as:

$$\alpha =\arctan(g^{\prime }/a_{f})\cdot 180/\pi$$

The equivalent slope ES was calculated as:

ES=tan((90−α)·π/180)

The equivalent mass EM was calculated as:

$$EM=g^{\prime }/9.81$$

The ($COT_{acc}$) was calculated based on the same formulas as for the estimation of $COT_{elev}$ using the equivalent slope (ES):

$$Acceleration:\;COT_{acc}=1.561\cdot ES\cdot EM$$

$$Deceleration:\;COT_{acc}=\left(1.561\cdot ES+9.762\cdot ES^{2}+14.0\cdot ES^{3}\right)\cdot EM$$

The instantaneous COT was calculated for each second of the cross-country phase from continuously recorded GPS data. The cumulative COT was subsequently calculated for each ride by means of summation and then converted from $\mathrm{mL}\;O_{2}\cdot {\mathrm{kg}}^{-1}$ to $J\cdot {\mathrm{kg}}^{-1}$ by multiplication with the energy equivalent of oxygen ($20.1\;J/\mathrm{mL}\;O_{2}$). The power demand (PD) in $J\cdot {\mathrm{kg}}^{-1}\cdot s^{-1}$ or $W\cdot {\mathrm{kg}}^{-1}$ was calculated by dividing the cumulative COT by the total duration of the cross-country ride.

#### 2.3.3. Estimating the Aerobic and Anaerobic Energy Expenditure from Heart Rate and Post-Exercise Blood Lactate Concentration

In order to evaluate the estimation of energy demand based on GPS and course data by the proposed model, the actual energy consumption during cross-country competitions was additionally calculated from heart rate (HR) and blood lactate concentration (LAC). The aerobic component of energy expenditure $VO_{2}$ was estimated from heart rate according to [10].

$$VO_{2}\left(\mathrm{mL}\cdot {\mathrm{kg}}^{-1}\cdot \min^{-1}\right)=0.002816\cdot HR^{1.9955}$$

Although no universally accepted method exists for determining the anaerobic contribution, an increase of 1 mmol/L in blood lactate is estimated to correspond to approximately 3 mL of oxygen per 1 kg of body mass, based on empirical data from human studies. The validity of this conversion has been demonstrated across a range of exercise types, intensities, and individuals [23]. The rate of blood lactate accumulation was estimated by dividing the blood lactate concentration measured at 10 min post-exercise by the duration of exercise and converted to oxygen consumption by the aforementioned conversion factor. Total power output (PO) was calculated by summation of aerobic and anaerobic components of oxygen consumption and converting oxygen consumption in $\mathrm{mL}\cdot {\mathrm{kg}}^{-1}\cdot \min^{-1}$ to $W\cdot {\mathrm{kg}}^{-1}$, as described above.

### 2.4. Statistical Analysis

A linear mixed-effects model (LMM) was established, with the power output estimated from heart rate and blood lactate concentration (PO) serving as the response variable and the $PD_{path}$, $PD_{acc}$, and $PD_{elev}$ as the fixed effects. This model was formulated using the ‘lmer’ function in R version 4.4.3 [24]. The majority of equines were sampled on multiple occasions, with some subjects being sampled more frequently than others. Consequently, the contributions made by each horse to the data set were not uniform, and the independence of observations could not be assumed. In order to account for multiple measurements of the same horse on different occasions and of several horses on the same occasion, the horse, as well as the event nested within the venue, were included as random intercepts. The model incorporated additional fixed-effects variables, namely the level and format of the cross-country competition, as well as the amount of thoroughbred blood in the horses. These variables have previously been shown to significantly influence heart rate and blood lactate concentration in response to cross-country tests [5].

## 3. Results

### 3.1. Data

A summary of the data subset that has been used to develop the energetic cost model can be seen in Table 1.

### 3.2. Model

The relationship between power output estimated by heart rate and blood lactate concentration and power demand estimated by GPS and course data is described by the following model:

$$PO=1.31\cdot PD_{path}+0.59\cdot PD_{acc}+0.48\cdot PD_{elev}-3.67\cdot TB+8.17$$

The marginal $R^{2}$ was 0.29, the conditional $R^{2}$ was 0.91. The intraclass correlation coefficient (ICC) was 0.77 for horses and 0.10 for event nested within venue.

**Figure 1:**
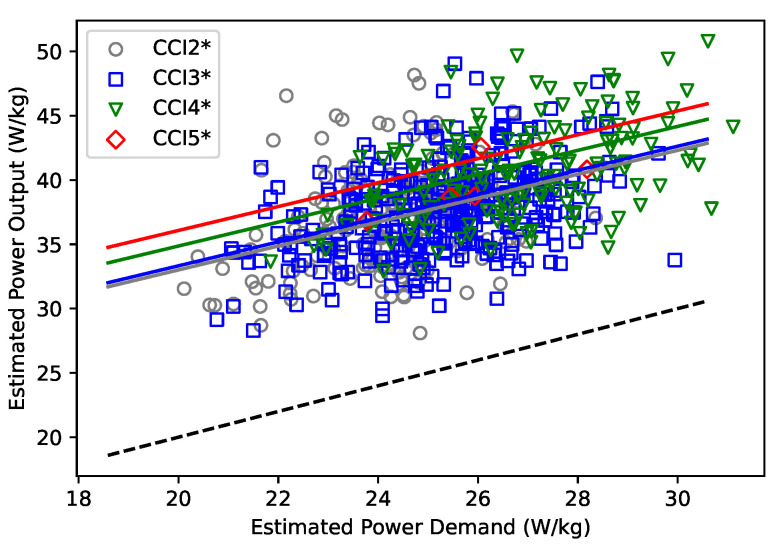
Power output estimated from 256 horses during 691 cross-country competitions on 2-star (CCI2*), 3-star (CCI3*), 4-star (CCI4*) and 5-star (CCI5*) level based on the power demand calculated from $PD_{path}$, $PD_{acc}$, and $PD_{elev}$. Line of identity (dotted) and regression lines of the linear mixed-effects model are shown separately for different competition levels.

The power output estimated by the LMM in relation to the estimated power demand is shown in Figure 1. The estimated power output was affected by $PD_{path}$ ($\chi^{2}$ (1) = 157.9, *p* < 0.001), $PD_{acc}$ ($\chi^{2}$ (1) = 16.6, *p* < 0.001), $PD_{elev}$ ($\chi^{2}$ (1) = 10.7, *p* < 0.01), competition level ($\chi^{2}$ (3) = 36.9, *p* < 0.001), and proportion of thoroughbred blood ($\chi^{2}$ (1) = 15.7, *p* < 0.001) but not by competition format ($\chi^{2}$ (1) = 2.5, *p* = 0.11).

**Figure 2:**
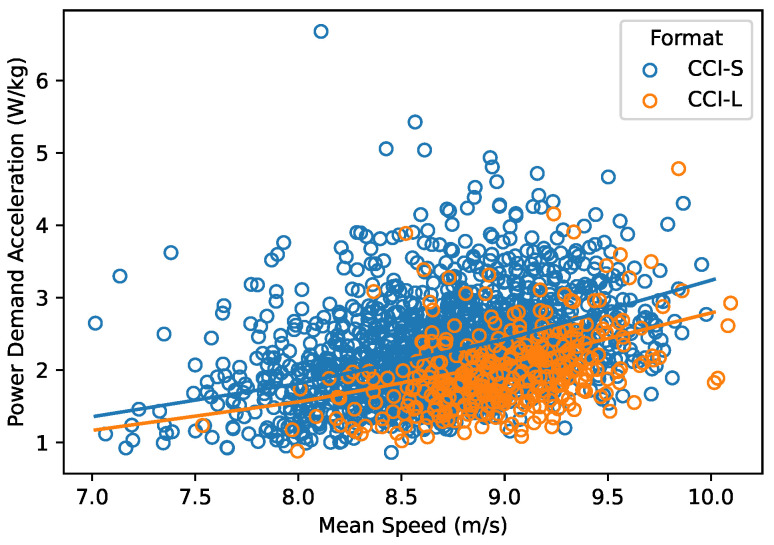
$PD_{acc}$ estimated from 394 horses during 1978 cross-country competitions in relation to mean speed. Regression lines of the linear mixed effects model are shown separately for short (CCI-S) and long (CCI-L) competition formats.

The $PD_{acc}$ increased with increasing mean speed ($\chi^{2}$ (1) = 426.1, *p* < 0.001), as shown in Figure 2. In short format competitions, $PD_{acc}$ was higher than in long format competitions at the same speed ($\chi^{2}$ (1) = 52.0, *p* < 0.001).

**Figure 3:**
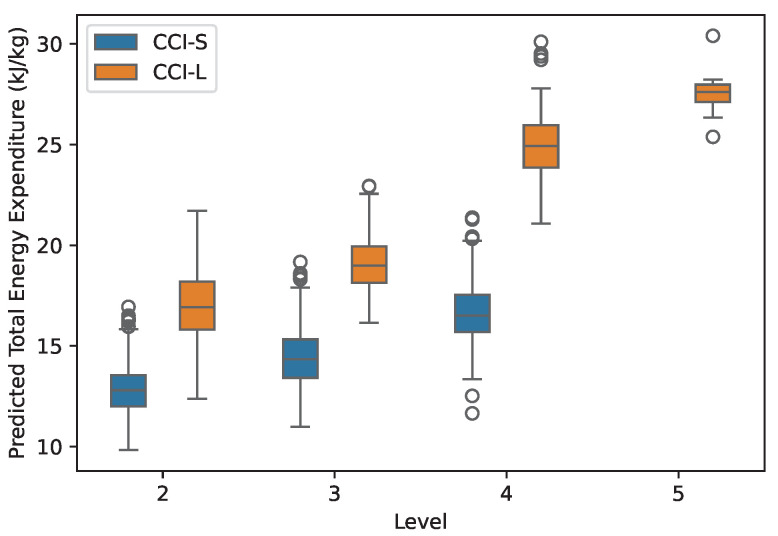
Total energy expenditure predicted by the proposed model from GPS data of 394 horses during 1978 cross-country competitions in relation to competition level (2-star to 5-star) and format (short: CCI-S; long: CCI-L).

**Figure 4:**
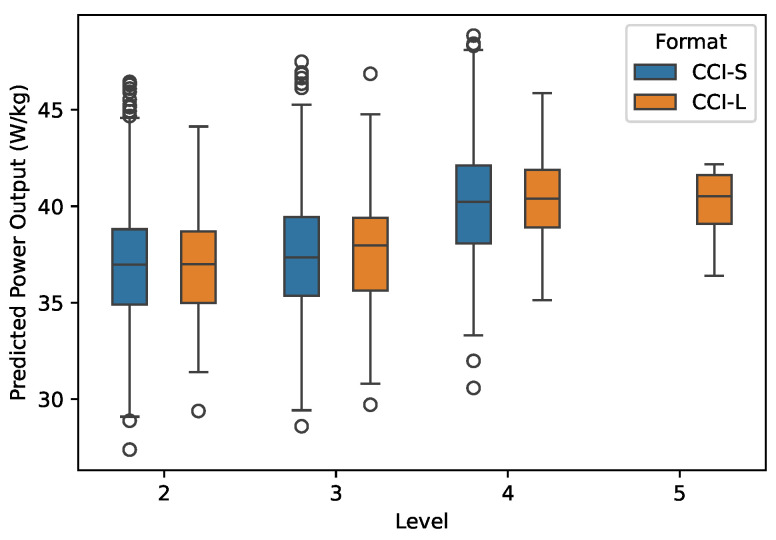
Power output predicted by the proposed model from GPS data of 394 horses during 1978 cross-country competitions in relation to competition level (2-star to 5-star) and format (short: CCI-S; long: CCI-L).

The total energy expenditure in $\mathrm{kJ}\cdot {\mathrm{kg}}^{-1}$ for the entire duration of the cross-country tests and the power output in $W\cdot {\mathrm{kg}}^{-1}$ predicted by the proposed model are shown in Figure 3 and Figure 4.

### 3.3. Anaerobic Contribution to Total Power Output

**Figure 5:**
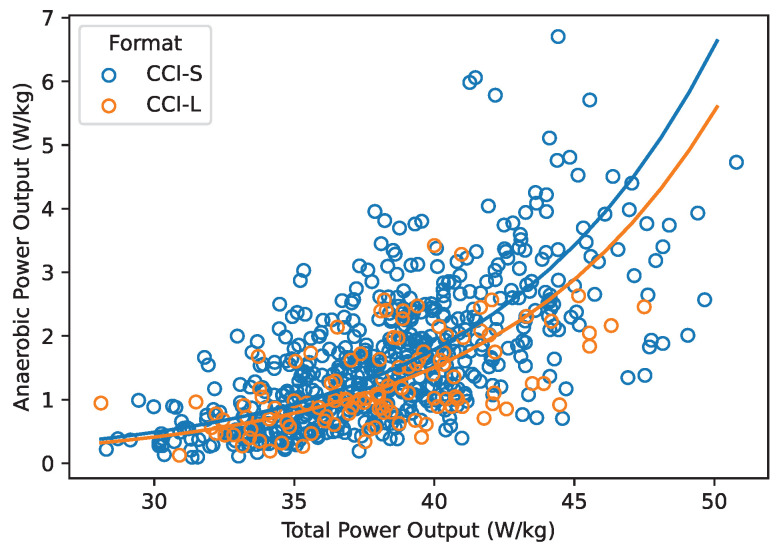
Anaerobic power output estimated from 256 horses during 691 cross-country competitions in relation to total power output. Regression lines of the linear mixed effects model are shown separately for short (CCI-S) and long (CCI-L) competition formats.

The anaerobic contribution to total power output during cross-country tests increased with increasing total estimated power output ($\chi^{2}$ (1) = 342.0, *p* < 0.001) as shown in Figure 5. Short format competitions resulted in higher anaerobic power output in relation to total power output of long format competitions ($\chi^{2}$ (1) = 8.5, *p* < 0.01). In contrast, the competition level did not affect anaerobic contribution to power output ($\chi^{2}$ (1) = 3.5, *p* = 0.32).

**Figure 6:**
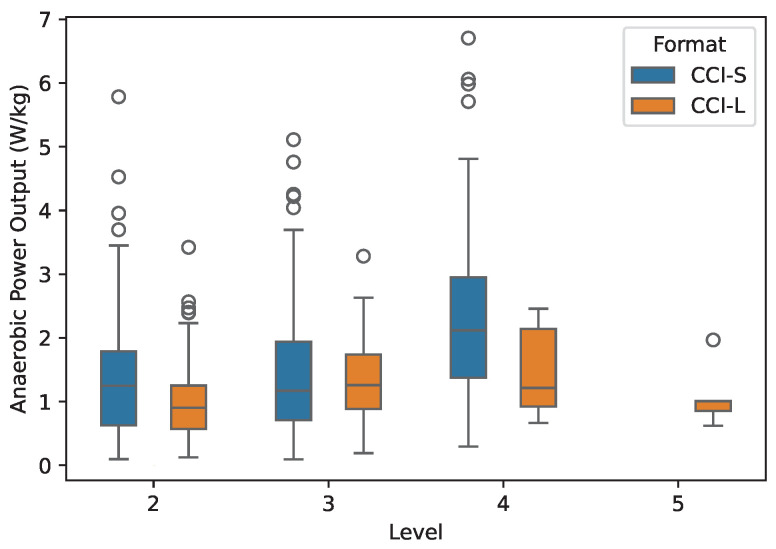
Anaerobic power output estimated from 256 horses during 691 cross-country competitions in relation to competition level (2-star to 5-star) and format (short: CCI-S; long: CCI-L).

The anaerobic contribution to total power output for different classes is shown in Figure 6.

## 4. Discussion

The cost of transport for path ($COT_{path}$), acceleration ($COT_{acc}$), and elevation ($COT_{elev}$) were found to have a significant effect on energy expenditure during the cross-country phase, as estimated from heart rate and blood lactate concentrations. The findings of this study indicate that, in addition to average speed and terrain, variability in speed, that is, fluctuations in pace throughout the course, exerts a substantial influence on the total energy expended by the horse. It is imperative that this variability is explicitly accounted for when modelling the energy demands of cross-country courses. Speed fluctuations may be influenced by the design of the course, but they are also likely to reflect the experience, skill, and training status of both horse and rider. A rider capable of guiding the horse through the course with minimal pace variation may significantly reduce energetic costs. This phenomenon appears to be especially pertinent in the context of short format competitions and at elevated speeds. The findings suggest that energy demand, attributable to fluctuations in speed, exhibits a marked increase with increasing speeds, being particularly pronounced in short format competitions.

A significant overall intercept between COT and energy expenditure estimated from heart rate and blood lactate concentration of approximately 8 $W\cdot {\mathrm{kg}}^{-1}$ was observed. This indicates that the energy expenditure during cross-country courses is underestimated by the calculated COT alone. This finding underscores the existence of hitherto unexplored influential factors that have not been incorporated into the existing model.

The level of competition exhibited a substantial influence on energy expenditure, despite the fact that disparities in mean velocity and acceleration were already encompassed within the COT components. This finding suggests that competition level introduces further energetic demands, likely due to the increased size, frequency, and technical difficulty of jumps at higher levels. However, it should be noted that the impact of the competitive level was found to be relatively small.

In order to assess the performance of the model, both marginal and conditional R² values were examined. The conditional R², which reflects the variance explained by both fixed and random effects, indicated that the model accounted for approximately 90% of the variance in energy expenditure. Conversely, the marginal R², which represents solely the fixed effects (i.e., the estimated cost of transport), accounted for approximately 30% of the variance. This substantial difference underscores the significance of individual and event-level variability that is not solely attributable to the cost of transport. The intraclass correlation coefficients, which provide a quantitative measure of the proportion of variability explained by different random effects, further indicate that the variability explained by differences between horses is considerably higher than that explained by differences between events. This finding serves to further highlight the relevance of individual differences between horses.

Between-event variability may be attributed to environmental factors, including footing quality, ambient temperature, humidity, and course layout. These factors were not incorporated into the present model. Furthermore, the seasonal timing of a competition has the potential to influence equine fitness levels; for instance, performance may exhibit an upward trend as horses become more fit over the course of the season or a downward trend as a result of accumulated fatigue.

It is highly likely that differences in individual fitness levels are a primary contributing factor to between-subject variability. This fitness level is determined by a combination of genetic predisposition and the status of the horse’s training. Although variables such as age and experience could potentially serve as proxies for training status, they are not universally reliable. For example, horses may return after extended breaks due to injury. Although the present study did not directly quantify individual training status, the proposed model offers valuable potential for supporting individualised training strategies, monitoring fitness, and managing risk in eventing horses. By assessing relative exercise intensities through heart rate and post-exercise blood lactate concentrations in individual horses and comparing them to predicted exercise intensity derived from a reference population based on external factors such as speed and terrain, information about the fitness levels of individual horses may be provided. Horses exhibiting disproportionately high heart rate and blood lactate responses relative to the modelled energy demand may be less fit than the average competitor at the same level. The more accurately external workload parameters can be modelled during cross-country competitions, the more precisely individual fitness levels can be assessed. Furthermore, estimating the total energy demands of competitive efforts, along with the relative contributions of aerobic and anaerobic metabolism, is essential for designing targeted conditioning programmes. Such predictions also support informed decision-making regarding a horse’s physiological readiness to participate in a specific event.

Horses possess an exceptionally large aerobic capacity, with maximal oxygen consumption in untrained horses typically ranging from 80 to 140 $\mathrm{mL}\cdot {\mathrm{kg}}^{-1}\cdot \min^{-1}$). Evans [9] posited that training can increase this capacity by approximately 10–25%. The proposed model has been shown to estimate the range of aerobic power outputs during cross-country competitions. This range corresponds to oxygen consumption levels of approximately 80 to 140 $\mathrm{mL}\cdot {\mathrm{kg}}^{-1}\cdot \min^{-1}$. The variation in workload demands that is potentially attributable to training is, therefore, substantial.

While much of the current research on physiological effort markers in eventing horses focuses on elite-level athletes, it is important to recognise that horses with varying levels of fitness are regularly trained and compete across all levels of equestrian sports. As the present study was based on a large dataset, including horses competing from preliminary to elite level, the results should be representative not only of horses competing at the top level but of a relatively broad population of professional equine athletes, although not necessarily for amateur athletes. Several studies have demonstrated that less fit horses exhibit higher heart rates, greater blood lactate accumulation, and slower recovery after comparable workloads. For instance, ref. [25] observed that averagely conditioned horses reached lactate thresholds at significantly lower speeds than well-trained horses, suggesting a limited aerobic capacity and earlier reliance on anaerobic metabolism. These findings underscore the relevance of assessing physiological responses in horses of varying fitness levels, supporting the broader applicability of models like the one developed in this study, not only for elite competitors but also for managing training, recovery, and performance in amateur or developing sport horses.

Lastly, the estimated power output derived from physiological indicators could not be fully explained by the estimated mechanical power demand based on the parameters of $COT_{path}$, $COT_{acc}$, and $COT_{elev}$. This finding indicates that additional factors must be incorporated. These include technical aspects of the course, such as the number, type, and arrangement of jumps, features that are somewhat unique to a given course designer, all of which likely contribute to the total energetic cost and should be considered in future iterations of the model. A potential limitation of the proposed model is its basis on several assumptions and estimations. The estimation of energy expenditure from heart rate and blood lactate, based on empirically derived formulas, can only provide approximate values. Moreover, the rate of lactate accumulation was estimated from blood lactate concentrations measured 10 min after the cessation of exercise, which may have led to an underestimation of peak lactate concentrations due to lactate clearance between the termination of exercise and blood sampling. A further significant constraining factor pertains to the incorporation criteria. Specifically, the data points were incorporated into the model solely when all three sources, GPS, heart rate, and lactate, were present. This results in substantial variations in the n numbers across the various levels (see Table 1). A substantial increase in the diversity of the equine population was observed in the short format courses. This was due to an increase in the frequency and availability of these courses, which enabled the testing of a greater number of horses. The courses also included combinations of horse-rider pairs with varying levels of experience, ranging from inexperienced riders with inexperienced horses to experienced riders with inexperienced horses, to inexperienced riders with experienced horses, as well as expert riders with expert horses. As previously stated, a pivotal subsequent action would be to quantify the training and experience status of the horse-rider pair and incorporate these into the model. As demonstrated in Table 1, the long format courses, particularly the 5-star level, are not an accurate representation of the population. This is due to the fact that only a select few horse-rider combinations capable of competing at that level and fit to do so were sampled.

## 5. Conclusions

This study highlights that while key contributors to external load, namely speed, speed variability, and elevation change, can be quantified using commercially available tools, essential parameters are still missing from the model. As a result, the internal load required to complete a cross-country course cannot yet be fully explained. Although increased competition difficulty has a measurable effect, its contribution to energy expenditure is relatively small, suggesting the influence of other unmeasured variables.

One such variable is likely the heterogeneity within competition levels due to differences in course design and variation in horse-rider experience. These may obscure the specific energetic demands attributed solely to duration, speed, or obstacle height. Another important factor contributing to large between-subject variability is the individual fitness level of the horses. To improve model accuracy, additional descriptors such as horse signalment (e.g., age, breed), training status of horse and rider, and course designer identity should be incorporated. Despite the current limitations, this study demonstrates the feasibility of estimating cost parameters relevant to cross-country performance using accessible data sources. Future work should focus on refining model components and segmenting the dataset by horse-rider experience and training status. 

## Figures and Tables

**Table 1:** Summary of the data subset used to develop the energetic cost model shown separately for different competition star levels (CCI2* to CCI5*) and short (CCI-S) and long (CCI-L) formats. The number of horses (n_H_), cross-country rides (n_XC_), events (n_E_), and venues (n_V_) included are given. Distance, duration, mean speed, mean heart rate (HR), and blood lactate concentration measured 10 min post-exercise (LAC_10_) are given as the mean ± standard deviation.

Class	n_H_	n_XC_	n_E_	n_V_	Distance (m)	Duration (min)	Mean Speed (m/s)	Mean HR (b/min)	LAC_10_ (mmol/L)
CCI2*-L	44	48	19	8	4080 ± 250	7.7 ± 0.5	8.8 ± 0.2	197 ± 9	8.0 ± 5.0
CCI2*-S	106	141	49	17	2937 ± 156	5.8 ± 0.4	8.5 ± 0.4	196 ± 10	7.9 ± 5.5
CCI3*-L	40	51	21	8	4555 ± 130	8.4 ± 0.4	9.0 ± 0.3	199 ± 8	11.4 ± 5.4
CCI3*-S	146	286	79	24	3356 ± 194	6.4 ± 0.4	8.8 ± 0.4	198 ± 9	8.9 ± 5.8
CCI4*-L	13	14	7	3	5814 ± 351	10.4 ± 0.5	9.3 ± 0.3	206 ± 7	15.3 ± 7.3
CCI4*-S	83	146	52	12	3724 ± 194	6.8 ± 0.4	9.1 ± 0.4	203 ± 8	15.5 ± 7.8
CCI5*-L	5	5	5	2	6394 ± 163	11.6 ± 0.5	9.2 ± 0.2	204 ± 6	12.4 ± 5.4

## Data Availability

The datasets presented in this article are not readily available because the data were collected as part of the performance diagnostic services offered by the German Olympic Equestrian Committee and federally funded by the German government for the advancement of national sport. Requests for access to the datasets should be directed to the primary author.

## Abbreviations

The following abbreviations are used in this manuscript:

COT	cost of transport
HR	heart rate
ES	equivalent slope
EM	equivalent mass
PO	power output
PD	power demand
EE	energy expenditure
GPS	global positioning sattelite
FEI	Fédération Équestre Internationale
